# Bath-related headache: a case report

**DOI:** 10.1186/s13256-023-03960-8

**Published:** 2023-06-04

**Authors:** Thashi Chang

**Affiliations:** Department of Clinical Medicine, Faculty of Medicine, University of Colombo, 25, Kynsey Road, Colombo, 00800 Sri Lanka

**Keywords:** Hot water, Asian, Thunderclap, Headache

## Abstract

**Background:**

Bath-related headache (BRH) is a rare primary headache disorder with only about 50 cases reported from 2000 to 2017 and none since. It is an abrupt onset excruciating headache occurring predominantly in middle-aged Asian women, most commonly following exposure to hot water. This is the first report in a Sri Lankan woman.

**Case presentation:**

A 60-year-old Sri Lankan woman presented with an abrupt onset, severe throbbing holocephalic headache immediately following a hot-water shower. The headache was not associated with photo- or phonophobia, nausea, or vomiting, and she did not report a past history of migraine. However, she had experienced a similar headache 2 years previously precipitated by a hot-water shower. Her neurological examination, blood investigations, and magnetic resonance imaging of brain and intracranial vessels were normal. She was treated with opioid and nonsteroidal antiinflammatory drug analgesics, but the headache resolved only after treatment with nimodipine. The headache did not recur during a follow-up of 2 years since she avoided hot-water showers.

**Conclusions:**

Bath-related headache is a thunderclap primary headache disorder with a benign prognosis, but its recognition requires awareness to differentiate it from subarachnoid hemorrhage. It warrants inclusion in the International Classification of Headache Disorders.

## Introduction

Bath-related headache (BRH) is a rare headache disorder first described in the year 2000 [1] and found to predominate among Asian populations, with a high prevalence among women [2]. A review of all cases of BRH between 2000 and 2017 identified 50 reports from Taiwan (26), China (6), Japan (5), Turkey (4), India (3), Brazil (3), South Korea (1), Spain (1), and France (1) [3]. Since 2017, there has not been a single report in the English scientific literature. This is a report of the first case of BRH from Sri Lanka, revisiting the clinical features that characterize it and discussing its pathophysiology and treatment.

## Case presentation

A 60-year-old Sri Lankan woman residing in the suburbs of Colombo developed a severe headache immediately after a hot-water shower, which was bilateral and throbbing in nature but subsided after 30 minutes. The next day, she experienced a relapse of the headache, again after a hot-water shower, but since the headache persisted beyond an hour and was excruciatingly unbearable, she was admitted to hospital. The headache was not associated with nausea, vomiting, or photo- or phonophobia. She had experienced a similar headache 2 years previously when she had been in the central hill country area of Sri Lanka that has a cool climate, again precipitated after a hot-water shower. At that time, she had been admitted to the regional hospital and had a computerized tomography (CT) scan of the brain, which was normal. The headache had subsided after a few hours with analgesic medication. Since then, she had not had a hot-water shower until a hot-water geezer had been recently installed in her home. Her past medical history included dyslipidemia and osteoporosis, but no migraine or any other form of headache syndrome. She was on a replacement dose of thyroxine following thyroidectomy. She had a significant family history of metastatic breast and ovarian carcinoma.

**Fig. 1:**
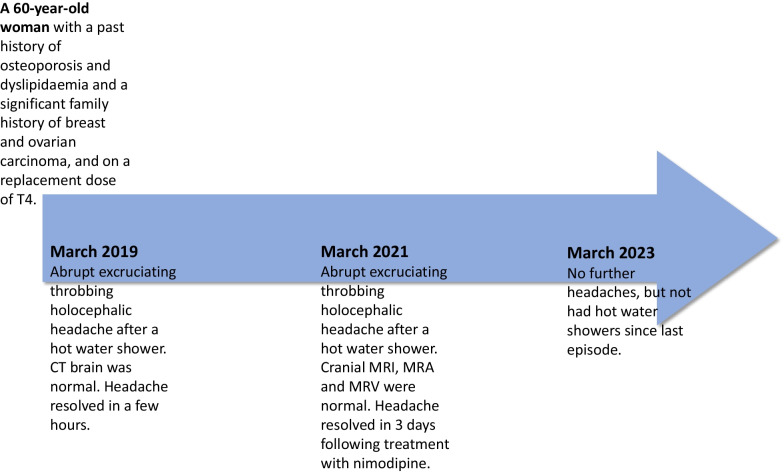
Timeline of the patient’s clinical course

Her neurological and general examinations were unremarkable. Her pulse was 72 beats per minute and her blood pressure was 140/90 mmHg. Hematological and biochemical investigations including inflammatory markers were within normal limits. Magnetic resonance (MR) imaging of the brain including MR angiography and MR venography were normal. She was treated with intramuscular pethidine and nonsteroidal antiinflammatory drug (NSAID) analgesics because of the severity of her headache, but the intensity dampened only after commencing nimodipine 60 mg/4 hourly, which was continued for 3 weeks. The headache continued as a dull ache for 3 days after commencing nimodipine. She was not prescribed any long-term medications for the headache. During the follow-up of over 2 years, she had not had a relapse and significantly, she had not had a hot-water shower since her last headache. The timeline of the patient’s clinical course is shown in Fig. 1.

## Discussion and conclusions

Bath-related headache (BRH) is a rare, primary headache disorder that presents as a thunderclap headache, but has a distinct trigger, which is the external contact of hot water, most commonly to the head. Occasionally, it may be triggered by cold water and contact of water to other body areas [4]. The triggers include taking a shower, soaking in a hot bath, diving into a swimming pool, exposure to steam, brushing of teeth, and rinsing of the mouth [2–4]. From the cases reported to date, direct skin stimulation by water and temperature changes rather than hot-water baths per se appears to be necessary components of the trigger. Thus, “thermic water-contact headache” may be a more accurate description than BRH.

The headache has a thunderclap nature similar to a subarachnoid hemorrhage, with an abrupt onset and a crescendo within minutes to reach its peak of excruciating explosive or throbbing holocephalic headache [2–4]. Although BRH fulfills the diagnostic criteria of a primary thunderclap headache, the International Classification of Headache Disorders (4.4 of ICHD-3) recommends that when a unique trigger is identifiable, such headaches should be coded according to its trigger [5]. However, BRH is yet to be included in the ICHD. The headaches usually self-limit within days to weeks and have a benign prognosis, albeit recurring with exposure to the trigger. However, posterior reversible encephalopathy syndrome has been rarely reported in BRH [6]. In about a third of patients, BRH may be associated with other primary headache disorders such as migraine and tension-type headache [3]. Why women are almost exclusively affected and the preponderance among Asian populations have been hypothesized to be related to fluctuations or deficiencies in sex hormones and a genetic predisposition [6].

The onset of BRH is most commonly in the fifth to seventh decade of life [2–4], as was seen in our patient. She had not experienced any form of primary headache disorder previously and was severely afflicted by the BRH when it occurred. Brain imaging including angiography is usually performed in these patients because of the differential diagnosis of subarachnoid hemorrhage. In one of the largest series of BRH (*N* = 21), multisegmental vasoconstriction, particularly of the middle and posterior cerebral arteries were noted in 62% of patients, suggesting that some of the BRH are due to reversible cerebral vasoconstriction syndrome [6]. However, the exact pathophysiology of BRH remains unclear, with patients demonstrating no abnormalities on intracranial vessel imaging as was the case in our patient. Thus, BRH remains a diagnosis of exclusion including the absence of cerebral vasoconstriction on intracranial vessel imaging.

Nimodipine, a calcium channel blocker used in the treatment and prevention of vasospasms, has been found to be effective in BRH without demonstrable intracranial vasospasm [7]. Anecdotal reports of NSAIDs and triptans as abortive therapy, and amitriptyline, gabapentin, sodium valproate, and topiramate as preventive therapy have not proven to be consistently effective [2] and are confounded by the high rate of spontaneous remission of BRH. Thus, no effective prophylactic therapy for BRH has been recognized except avoiding the trigger. Although the strategy of avoiding hot-water showers proved effective and doable in our patient living in a tropical country, it may not be practical for patients living in cooler climes. Occasionally, changing the method of exposure to hot water from a shower to immersion in a tub has been reported to be effective, while in others BRH had spontaneously resolved [4].

The intention of this case report is to increase awareness of this entity that needs to be differentiated from subarachnoid hemorrhage, and to add to the number of reports that would substantiate its inclusion in to the ICHD.

## Data Availability

All necessary data and material are provided.

## Abbreviations

BRH: Bath-related headache
CT: Computerized tomography
ICHD: International Classification of Headache Disorders
MR: Magnetic resonance
NSAIDs: Nonsteroidal antiinflammatory drugs
